# Folk taxonomy and traditional uses of common bean (*Phaseolus vulgaris* L.) landraces by the sociolinguistic groups in the central region of the Republic of Benin

**DOI:** 10.1186/s13002-018-0251-6

**Published:** 2018-07-31

**Authors:** Laura Estelle Yêyinou Loko, Joelle Toffa, Arlette Adjatin, Ahouélété Joel Akpo, Azize Orobiyi, Alexandre Dansi

**Affiliations:** 1 Laboratory of Applied Entomology, Faculty of Sciences and Technology of Dassa (FAST-Dassa), National University of Sciences Technologies Engineering and Mathematics of Abomey (UNSTIM), BP 14, Dassa, Benin; 2 Laboratory of Biotechnology, Genetic Resources and Plant and Animal Breeding (BIORAVE), FAST-Dassa, UNSTIM, BP 14, Dassa, Benin

**Keywords:** Medicinal uses, Mystical-religious uses, Religious prohibitions, Traditional knowledge, Vernacular nomenclature

## Abstract

**Background:**

Common bean (*Phaseolus vulgaris* L.) is an important grain legume crop grown in the central region of the Republic of Benin. However, its production declined in recent years to the extent that its diversity is being threatened with extinction. Understanding the folk nomenclature and taxonomy, as well as use values that allow its maintenance in Beninese agricultural system, is a prerequisite to develop efficient strategies for its conservation. Knowing that each sociolinguistic group develop various uses and traditional knowledge for their crop genetic resources, we hypothesized that enhancement of farmers’ livelihood, thanks to the use values of common bean landraces, differ from one sociolinguistic group to another and contribute to their conservation in the traditional agriculture of central Benin.

**Methods:**

Hundred and one common bean producers belonging to seven sociolinguistic groups selected through 23 villages of the region under study were surveyed. Data were collected through participatory research appraisal tools and techniques (individual interviews and direct observation) using a semi-structured questionnaire. Folk nomenclature and taxonomy of common bean, local uses, and factors affecting them were investigated.

**Results:**

Across the seven sociolinguistic groups surveyed in the study area, five common bean generic names and 26 folk varieties corresponding to 12 landraces have been recorded. Folk nomenclature and taxonomy were mainly based on seeds’ coat color. The present study has revealed five common bean use values in the study area (food, medicinal, commercial, fodder, and mystic-religious), which are influenced by sociolinguistic groups. Leaves, roots, and seeds of three common bean folk varieties are used by surveyed farmers for disease treatment. Nine common bean folk varieties are considered by farmers as magical plants which have supernatural properties while several taboos for deities’ followers regarding Séssé landrace are inventoried across sociolinguistic groups. Level of education and age of respondents influence positively and significantly medicine and mystical-religious uses of common bean respectively while commercial use is positively influenced by age and negatively by gender.

**Conclusions:**

Traditional values associated with common bean landraces in the central region of the Republic of Benin increase their chance of survival in the farming systems. Socio-demographic characteristics of respondents which influence common bean use values must be taken into account in future programs of conservation. However, an assessment of diversity and analysis of distribution of extend of common bean landraces in the study area is a necessity for the development of an efficient strategy of conservation of this genetic resource.

## Background

Common bean (*Phaseolus vulgaris* L.) is a legume commonly grown in sub-Saharan Africa for food, cash, animals’ food, and as soil improver [1]. Beans are often considered as the “poor man’s meat” and consumed as seeds (mature or immature) as well as a vegetable (both leaves and pods) [2]. Nutritionists characterize it as a nearly perfect food because of its high protein content and generous amount of fibers, complex carbohydrates, and other dietary necessities [3]. In Benin, common bean is grown on small plots and exploited by local populations in different regions [4]. Its cultivation covers more than 121,485 ha with a total yield of 101,821 tons in 2016 [5]. Among the domesticated *Phaseolus* species, common bean is the most cultivated species in the central region of the Republic of Benin where it plays a fundamental role in family farming and feeding of the local population [6].

Cultivated mainly for their edible seeds, the varietal diversity of common bean in the central region of the Republic of Benin is conserved by several socio-cultural groups [6], which classify, name, and group their varieties using different folk taxonomy descriptors [7]. However, vernacular names often have a very local distribution and may change with time because of incidental events and contact with other languages [8]. So the knowledge of folk nomenclature and taxonomy is very useful for communicating about common bean usage in local communities. Unfortunately, very little information exists on common bean folk taxonomy and nomenclature in Benin. While these pieces of information are vital for the development of in situ conservation scheme and help in developing seed distribution, flow networks, and establishment of regional varietal map [7].

The production of common bean in the Republic of Benin seems to be experiencing a regression in recent years [1], to the point of being threatened with extinction in certain regions of the country [6]. Indeed, in the central region of the Republic of Benin, several common bean landraces are in threat of disappearance [6]. Knowing that understanding of the value of a landrace is a pre-requisite prior to deciding on any conservation strategies [9], and that the positive landrace use values plays an instrumental role in the promotion of its on-farm conservation [10], it is so important to understand use values of common bean allowing its maintenance in Beninese agriculture for the development of efficient strategies of conservation. Moreover, there exists a symbiotic relationship between biological diversity and cultural diversity [11], highlighting the importance to evaluate common bean uses at the community level. Indeed, understanding how a community uses a resource is crucial for developing a framework for its sustainable use [12].

It is known that dry beans are important sources of numerous nutrients and phytochemicals that protect against multiple diseases [13]. Indeed, some studies have shown that common bean treat diabetes [14] and obesity [15, 16]. Moreover, mystic-religious use of common bean was observed by Papp et al. [17] in Romania. Unfortunately, less is known on the pattern of the use of common bean landraces and how its use varies among sociolinguistic groups throughout central Benin. While understanding traditional knowledge of plant species is crucial not only to preserve this knowledge but also to orient management for sustainable usage [18]. Moreover, this will serve as a basis for further scientific study of this natural resource in order to develop new and improved drugs and remedies [19].

This study aimed to test the following hypotheses. Knowing that a knowledge of folk taxonomy helps to develop an in situ conservation scheme for farmers’ varieties [20] and farmers use their own common bean classification system, we hypothesized that naming and classification of landraces varies in function of sociolinguistic groups and reflect the diversity of this legume in the study area. Assuming that each sociolinguistic group develops various uses for their crops genetic resources [10], we hypothesized that the use values of common bean landraces differ from one sociolinguistic group to another and contribute to the maintenance of this legume in traditional agriculture of central Benin. Based on the evidence that socio-cultural factors influence the use value of a landrace [21], we assumed that gender and education of farmers determine the categories of common bean uses.

## Methods

### Study area

**Fig. 1:**
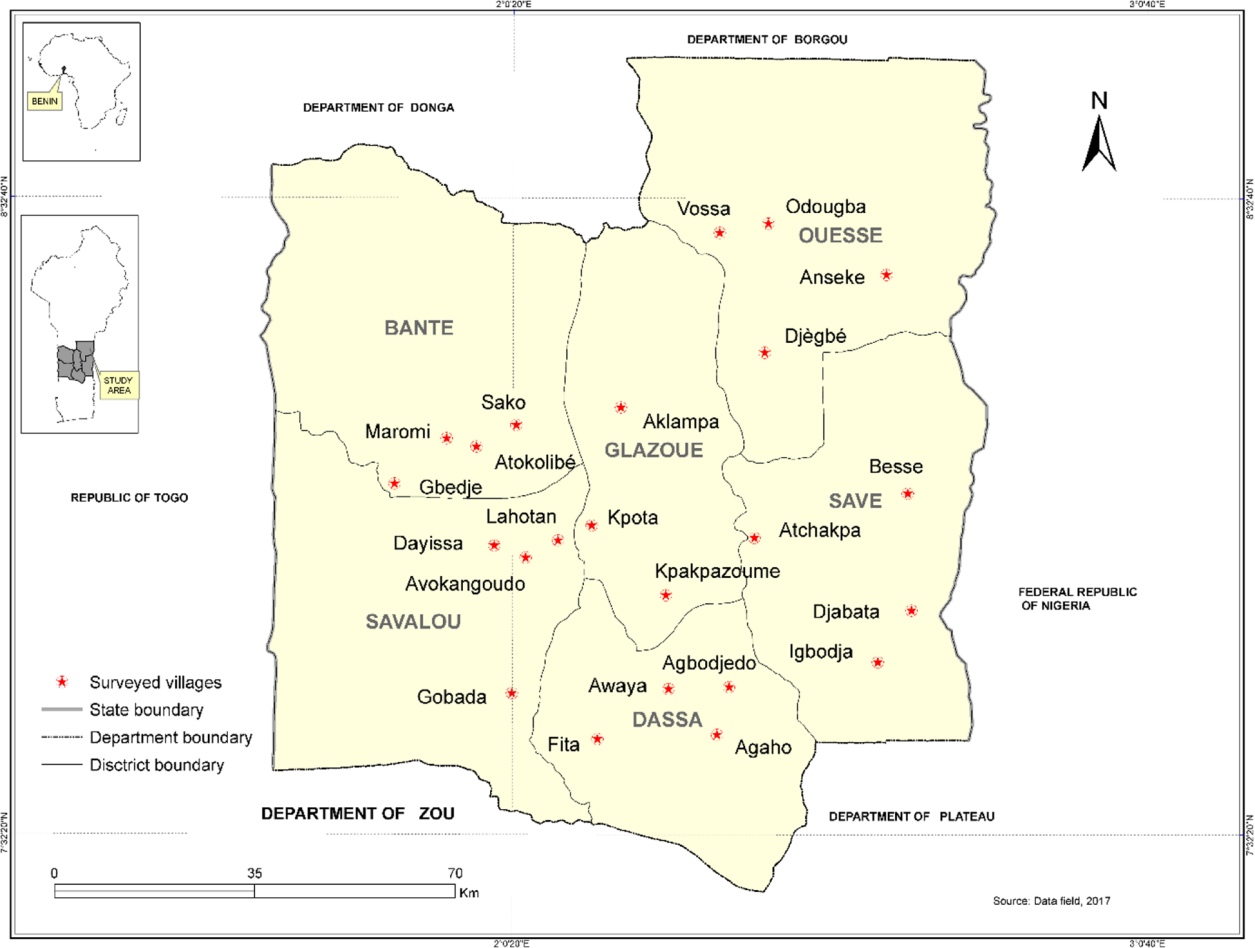
Map of Central Benin showing the geographical position of the surveyed villages

Covering an area of approximately 13,900 km^2^, Collines department in Benin is located between 7° 27′ and 8° 46′ north latitude and between 1° 39′ and 2° 44′ east longitude. It is a Sudano-Guinean climatic zone referring to the transition zone between the subequatorial and Sudanian zones. The study site has a rainfall regime straddle of bimodal distribution of the south and that of the unimodal distribution of the north. The annual rainfall varies between 900 mm and 1200 mm. Temperatures undergo great variations during the year and varies from 20 to 37 °C (Table 1). A variety of soils exists in the study area. The most important are the tropical ferruginous soils and hydromorphic soils. The natural vegetation consists of gallery forests along the drainage axes, open woods, and wooded savannahs on vertisols as well as saxicolous savannahs on the hills [22]. The population is estimated at 535, 923 inhabitants, and it is constituted by several socio-cultural groups, the majority of which are the Tchabe, Mahi, Idaasha, Fon, and related groups [23]. In each of the six municipalities (Bantè, Dassa-Zoumè, Glazoué, Ouèssè, Savalou, and Savè) making up the Collines department, 23 villages were chosen on the basis of two criteria such as the sociolinguistic groups and the common bean production (Fig. 1).

**Table 1:** Basic information regarding the seven sociolinguistic groups surveyed in the study area

	Municipalities of					
	Banté	Dassa-zoumé	Glazoué	Ouèssè	Savalou	Savé
Sociolinguistic groups surveyed	Nago, Ifé	Idaatcha, Fon	Mahi, Adja	Nago, Tchabè	Mahi, Idaatcha	Tchabè, Idaatcha
Population	107,181	112,122	124,431	142,017	144,549	87,177
Climate	Transition between subequatorial and Sudano-Guinean climate	Sub-equatorial climate	Sub-equatorial climate	Tropical climate intermediate between Guinean and Sudanese climate	Transition between subequatorial and Sudano-Guinean climate	Tropical climate intermediate between Guinean and Sudanese climate
Area (Km^2^)	2695	1711	1750	3200	2674	2228
Annual rainfall (mm)	600–1600	900–1100	959.56–1255.5	1100–1200	864–1637.3	1100–1300
Annual temperature (°C)	23 to 37	21 to 36	24 to 29	24 to 26	23 to 36	20 to 34
Vegetation	Wooded savannah area with part of classified forest	Wooded savannah and shrubby cut deciduous and semi-deciduous forests	The vegetation consists of natural formations	The plant cover is made of wooded savannah, shrubby, gallery forests and part of classified forest	The vegetation consists of islands of dense forest, savannah, fallow land and fields.	Graminaceous savannah with trees and shrubs. Classified forest
Soils	Tropical ferruginous soils	Tropical ferruginous soils	Soils sandy clay, hydromorphic and tropical ferruginous	Soils are clayey, hydromorphic and tropical ferruginous	Tropical ferruginous soils	Tropical ferruginous soils
Farming system	Cassava-Yam-Maize-based	Cassava-Soybean-Maize-based	Yam-Rice-Cassava-based	Cassava-Yam-Groundnut-based	Cassava-Yam-Maize-based	Cassava-Yam-Soybean-based
Number of surveyed villages	4	4	3	4	4	4

Data assembled from INSAE [77], Yabi et al. [78], and Akoegninou et al. [79]

### Data collection

In each of the selected villages, data were collected through semi-structured interviews and direct observation using a questionnaire [24]. Interviews were conducted with the help of local interpreters in each village to facilitate discussions with farmers [25]. In each village, individual interviews were made up with 5 to 7 common bean producers of both sexes and different ages, selected with the help of the village chief or farmers’ organization leaders and snow ball technique (where interviewed farmers own suggest their fellows to be interviewed). Before conducting the interviews, the objectives of the study were explained to the farmers in their respective local languages, and their consent was obtained [26]. A total of 101 households were interviewed through the seven sociolinguistic groups recorded in the study area. The Mahi sociolinguistic group was the most represented (31.7% of farmers), followed by Idaasha (21.8% of farmers), Fon (17.8% of farmers), Nago (10.9% of farmers), Ifé (7.9% of farmers), Tchabé (5.9% of farmers), and Adja (4% of farmers) sociolinguistic groups. The socio-demographic characteristics (name, gender affiliation, age of respondents, sociolinguistic group, education level, and number of years of experience in common bean production) of surveyed farmers were firstly collected. Men were the most surveyed farmers (72.3% of farmers), and this is across all sociolinguistic groups (Table 2). Most of the surveyed farmers are young with their age between 35 and 55 years. Through the sociolinguistic groups, a high rate of illiteracy was recorded in general (79.2%), surveyed farmers do not have formal education. The majority of surveyed farmers (59.4%) have 11 to 21 years of experience in common beans production. Similar tendencies were observed across sociolinguistic groups except the Nago and Ifé sociolinguistic groups, where most farmers have in majority 1 to 10 years of experience. Before interview, farmers were requested to bring samples of common bean folk varieties they cultivated or used. For each common bean folk varieties presented in local name by farmers, folk nomenclature and taxonomy, the use forms (seeds, stems, roots, and leaves), and the religious prohibitions were documented. When farmers listed one usage category of common bean folk varieties, we asked them to give us the use form, the preparation methods, and mode of application. After interview with each farmer, common bean folk varieties were collected and classified at laboratory using visual technique following Mohammed et al. [27] based on seed’s morphological description characteristics (coat color, size, coat pattern, and hilum color).

**Table 2:** Socio-demographic characteristics of the surveyed farmers in function of sociolinguistic groups

		Sociolinguistic groups							Total	Percentage
		Fon	Idaasha	Mahi	Nago	Ifé	Tchabé	Adja		
Gender	Men	13	12	27	8	5	5	3	73	72.3
	Women	5	10	5	3	3	1	1	28	27.7
Age	[35–56]	15	14	18	10	7	5	3	72	71.3
	[56–66]	3	7	11	0	0	0	1	22	21.8
	[66–76]	0	1	3	1	1	1	0	7	6.9
Education	No formal education	14	18	26	8	5	6	3	80	79.2
	Primary	3	2	5	2	3	0	1	16	15.8
	Secondary	1	2	1	1	0	0	0	5	5
Experience	[1–11]	7	4	6	8	6	1	2	34	33.7
	[11–22]	11	16	23	2	2	4	2	60	59.4
	[22–32]	0	2	3	1	0	1	0	7	6.9

### Data analysis

The data obtained during the surveys were analyzed by descriptive statistics (mean, percentage, variance, etc.) using Microsoft Excel 2010 software to generate figures and tables. According to Koura et al. [28], interviewee diversity value (ID) and equitability value (IE) were calculated to measure how common bean use forms are distributed among the interviewees and the degree of homogeneity of the interviewee’s knowledge respectively following the formulas described by Byg and Baslev [29]:

$$ \mathrm{ID}=\frac{\mathrm{Number}\ \mathrm{of}\ \mathrm{uses}\ \mathrm{cited}\ \mathrm{by}\ \mathrm{a}\ \mathrm{given}\ \mathrm{interviewee}\ }{\mathrm{Total}\ \mathrm{number}\ \mathrm{of}\ \mathrm{uses}};\mathrm{IE}\kern0.5em \frac{\mathrm{Interviewee}\ \mathrm{diversity}\ \mathrm{value}\ \left(\mathrm{ID}\right)}{\mathrm{Inde}{\mathrm{x}}^{\prime}\mathrm{s}\ \mathrm{max}\mathrm{imum}\ \mathrm{value}\mathrm{s}\ \left(\mathrm{ID}\mathrm{max}\right)} $$

Similarly, to measure the importance of the common bean use categories and the degree of homogeneity of knowledge about use categories in function of sociolinguistic groups, the use diversity value (UD) and use equitability value (UE) were calculated according to Koura et al. [28] following the formulas described by Byg and Baslev [29]:

$$ \mathrm{UD}=\frac{\mathrm{Number}\ \mathrm{of}\ \mathrm{indications}\ \mathrm{recorded}\ \mathrm{by}\ \mathrm{category}}{\mathrm{Total}\ \mathrm{number}\ \mathrm{of}\ \mathrm{indications}\ \mathrm{for}\ \mathrm{all}\ \mathrm{categories}};\mathrm{UE}\kern0.5em \frac{\mathrm{Use}-\mathrm{diversity}\ \mathrm{value}\ \left(\mathrm{UD}\right)}{\mathrm{Inde}{\mathrm{x}}^{\prime}\mathrm{s}\ \mathrm{max}\mathrm{imum}\ \mathrm{value}\mathrm{s}\ \left(\mathrm{UD}\mathrm{max}\right)} $$

To evaluate the differences of the ID, IE, UD, and UE indices related to sociolinguistic groups, the calculated indices were submitted to analysis of variance (ANOVA) after determination of data normality and homogeneity of variance. Significant differences between the means were separated using Student–Newman–Keuls statistic at the 5% level of probability. To describe the relationship between the use forms of common bean and the sociolinguistic group of the study area, data of use values ethnic groups were subjected to Principal Component Analysis (PCA) using Minitab 17 software.

Following Gouwakinnou et al. [30], the answer rates per specific use defined as the fidelity level (FL) in each study zone have been calculated as the ratio of number of informants related to a specific use by the total number of informants. This fidelity level was also used to calculate the use frequency of different plant parts [31].

The socio-demographic characteristics of surveyed farmers that affect the use of common bean in the central region of the Republic of Benin were analyzed using multinomial logic regression model. In this model, the dependent variable is multinomial with many categories that illustrate the diversity of the use of common bean inventoried in the study area. The specification of the empirical model or reduced form is as follows:

$$ \mathrm{yi}=\kern0.5em f\ \left(X1,X2,X3,X4\right) $$

Where “yi”, polychotomous dependent variable, is the common bean type of use made by farmers, and “*X*1 to *X*4” are the explanatory variables. Based on the diversity of the use of common bean by farmers in the study area, the dependent variable (yi) has been coded 1 for “food,” 2 for “medicinal,” 3 for “mystical-religious,” 4 for “commercial,” and 5 for “fodder.” Explanatory variables include: *X*1 = level of education, *X*2 = age, *X*3 = sex, and *X*4 = years of experience in common bean production. The estimation of the model of the multinomial logic regression was made considering the category “food” as the reference category.

## Results

### Folk nomenclature and taxonomy

Across the seven sociolinguistic groups surveyed in the study area, four common bean generic names in the local dialects were recorded: *Akpakoun* (Fon, Mahi, and Tchabé sociolinguistic groups), *Kpalakoun* or *Akpalakoun* (Nago, Ifé, and Idaasha sociolinguistic groups), and *Kpankoui* (Adja sociolinguistic group). A total of 26 common bean folk varieties were recorded. The majority of names given to common bean folk varieties (69.2%) have different meanings from their generic names (30.8%). The names assigned to common bean folk varieties corresponded mainly to seed coat color (90.7% of responses), growth habit (2.8% of responses), seed size (1.9% of responses), origin of folk varieties (0.9% of responses), perception of farmers on the magic (2.8% of responses), and agronomic (0.9% of responses) properties of folk varieties (Table 3). The identification of the different common bean folk varieties by farmers is based on the color of the seed coat (53.5% of farmers), the seeds shape (36.9% of farmers), the shine of seeds (8.3% of farmers), and the smell of seeds (1.3% of farmers).

**Table 3:** Meaning of the vernacular names of common bean folk varieties across sociolinguistic groups in the study area

Criteria of denomination	Percentage of responses	Naming of folk varieties	Sociolinguistic groups	Meaning of the vernacular name
Seed coat color	90.7	Akpakoun wéwé	Fon, Mahi	White bean
		Kpalakoun founfoun	Idaatcha, Tchabé	
		Akpakoun vovo	Fon, Mahi	Red bean
		Kpalakoun kpikpa	Idaatcha, Nago	
		Kpankoui rouge	Adja	
		Akpakoun kpikpa	Tchabé, Idaatcha	
		Akapakoun rouge	Mahi, Nago	
		Akpakoun wiwi	Fon	Black bean
		Ewoudjè	Tchabé	
		Sonouhoué	Mahi	Color of guinea fowl plumage
		Akpakoun sonhouékan	Fon	
Growth habit	2.8	Akpakoun djihikoun	Fon, Mahi	Bean from above
		Ewaarigui	Nago	Climbing bean
Magic properties	2.8	Kpankoui	Adja	Who seeks my evil will die
Seed size	1.9	Akpakoun wéwé winiwini	Fon, Mahi	White bean of very small size
		Akpakoun wéwé gaga	Fon, Mahi	White bean of big size
Origin	0.9	Mitoyikoun	Fon, Mahi	Bean of our ancestors
Agronomic properties	0.9	Sèkpavikoun,	Mahi	Bean that kills quackgrass

### Diversity of common bean landraces across sociolinguistic groups

Based on seed morphological characteristics, the 26 common bean folk varieties recorded, correspond, subject to synonymy, to 12 different landraces (Table 4). The number of common bean landraces varied from 3 to 8 in function of sociolinguistic groups (Table 5). Fon, Mahi, and Tchabé sociolinguistic groups presented the greatest number of common bean landraces (8), while Adja sociolinguistic group presented the smallest number of landraces (3). Subject to synonymy, *Séssé* landrace and the small red common bean locally called *Akpakoun vovo* (Fon and Mahi sociolinguistic groups), *Kpalakoun kpikpa* (Idaasha and Nago sociolinguistic groups), or *Kpankoui rouge* (Adja sociolinguistic group) were recorded through all sociolinguistic groups, except Adja and Ifé sociolinguistic groups respectively. Apart from *Akpakoun wiwi* landrace (small seed with black broad striped seed coat pattern and black color around hilum), which is only detained by the Fon sociolinguistic group, all remaining common bean landraces are shared by at least two sociolinguistic groups.

**Table 4:** List of landraces, their seed characteristics, and corresponding folk varieties according to sociolinguistic groups in the study area

No. of landrace	Seed’s morphological description	Folk varieties (sociolinguistic group)
1	Large flat seed with white seed coat color	- Akpakoun wéwé (Fon, Mahi) - Akpakoun wéwé gaga (Fon, Mahi) - Kpalagui (Ifè) - Kpalakoun founfoun (Idaatcha, Tchabé) - Kpakpalaegui (Adja, Nago)
2	Small shiny seed with red seed coat color	- Akpakoun vovo (Fon, Mahi) - Kpalakoun kpikpa (Idaatcha, Nago) - Kpankoui rouge (Adja) - Kpokpodo (Tchabè)
3	Small round seed with brown seed coat color and dark hilum color	- Séssé (Fon, Mahi, Idaatcha, Tchabè, Nago, Ifé)
4	Small flat seed with white seed coat color	- Akpalakoun founfoun (Idaatcha, Tchabè, Ifé) - Akpakoun wéwé winiwini (Fon, Mahi) - Kpankoui wéwé (Adja)
5	Small seed with marginal seed coat speckled of red and a red color around hilum	- Akpakoun sonhouékan (Fon) - Sèkpavikoun (Mahi) - Alawoaho (Tchabé)
6	Small seed with red broad striped seed coat pattern and red color around hilum	- Akapkoun rouge (Mahi, Nago)
7	Small seed with brown seed coat and red color around the hilum	- Akpakoun djihikoun (Fon, Mahi) - Ewaarigui (Nago)
8	Small seed with black broad striped seed coat pattern and black color around hilum	Akpakoun wiwi (Fon)
9	Large seed with black seed coat	- Sonouhoué (Mahi) - Ewoudjè (Tchabé) - Kpankoui wiwi (Adja)
10	Small flat shiny brown seeds with black color around hilum	Mitoyikoun (Fon, Mahi)
11	Large flat seed with red seed coat	Akpakoun kpikpa (Tchabé, Idaatcha)
12	Small white smooth seed with black color around hilum	Akpalakoun wéwé (Idaatcha, Ifé)

**Table 5:** Common bean landraces diversity in the seven sociolinguistic groups

Sociolinguistic groups	Number of landraces shared between sociolinguistic groups							Number of unique landraces	Total number of landraces
	Fon	Idaasha	Mahi	Nago	Ifé	Tchabé	Adja		
Fon	–							1	8
Idaasha	5	–						0	6
Mahi	7	5	–					0	9
Nago	4	3	5	–				0	5
Ifé	4	3	3	2	–			0	4
Tchabé	5	4	6	4	2	–		0	7
Adja	2	2	2	1	1	2	–	0	3

### Distribution of knowledge of common bean uses across sociolinguistic groups

Common beans are used by all surveyed farmers in the study area. The interviewee diversity value (ID) reached more than 0.50 only in Ifé and Adja sociolinguistic groups. However, the interviewee diversity value of Ifé sociolinguistic group was significantly different from others (*p* ≤ 0.05), showing the diversification of knowledge on common bean use forms in this sociolinguistic group (Table 6). Similar trends have been observed concerning interviewee equitability value (IE) (Table 6). However, also in the Nago, Adja, and Tchabé, sociolinguistic groups, the knowledge related to the use forms of common bean folk varieties was distributed homogeneously with high interviewee equitability value (> 0.50) (Table 6).

**Table 6:** Quantitative measurements of knowledge about common bean uses in function of sociolinguistic groups of the study area and use diversity value (UD) and equitability value (UE) according to various uses of common bean

Sociolinguistic groups	ID	IE	Common bean use categories							
			Medicinal		Commercial		Mystical-religious		Fodder	
			UD	UE	UD	UE	UD	UE	UD	UE
Fon	0.37 a	0.47 a	0.07	0.28	0.22	0.44	0.02	0.34	–	–
Idaasha	0.38 a	0.47 a	0.02	0.07	0.27	0.54	0.01	0.14	–	–
Mahi	0.31 a	0.38 a	0.00	0.03	0.15	0.31	0.01	0.19	0.03	0.06
Nago	0.40 a	0.50 a	0.02	0.09	0.18	0.36	0.01	0.17	0.25	0.50
Ifé	0.53 b	0.65 b	0.04	0.17	0.43	0.87	0.04	0.52	–	–
Tchabé	0.43 a	0.54 a	0.03	0.11	0.25	0.50	0.02	0.34	–	–
Adja	0.50 a	0.63 ab	0.11	0.42	–	–	0.02	0.26	0.14	0.27

*ID* interviewee diversity value, *IE* interviewee equitability value; means within the same rows followed by the same letter are not significantly different (*p* < 0.05)

### Use categories of common beans across sociolinguistic groups

**Fig. 2:**
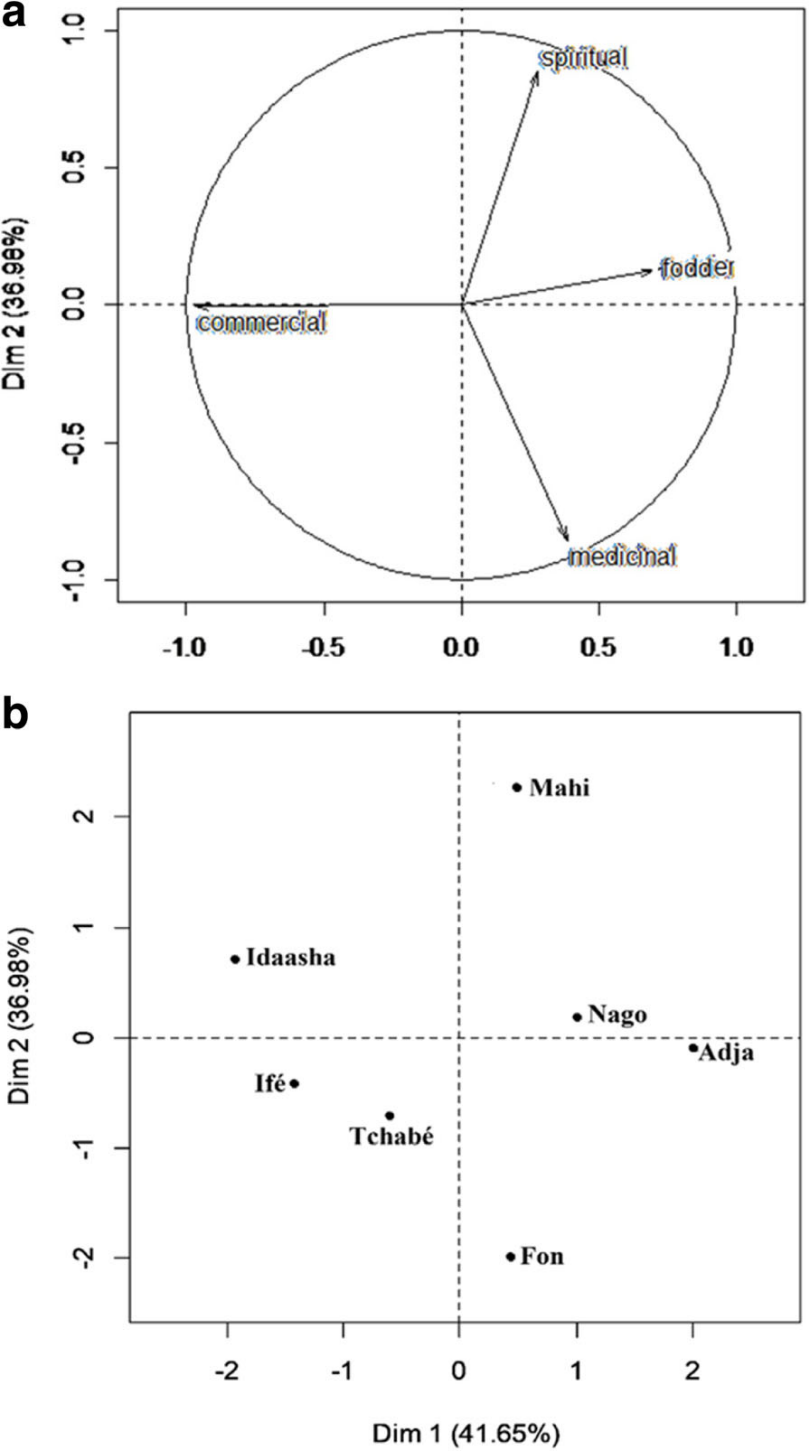
**a** Correlation circle of the plane formed by axes 1 and 2. **b** Projection of ethnic groups in the plane formed by the axes

Common bean folk varieties were widely used by sociolinguistic groups of central Benin. Five categories of the use of common bean folk varieties were recorded in central Benin. Indeed, the populations cultivate the species for food (48.8% of responses), commercial (22.4% of responses), medicinal (13.4% of responses), mystical-religious purposes (11.9% of responses), and fodder for sheep and goats (3.5% of responses). Apart from the food use (consumption of seeds alone or mixed to rice locally called *Atassi* or transformation of the seeds in donuts locally called *Ikalé*) that remains common to all sociolinguistic groups in the study area, a principal component analysis (PCA) has made it possible to determine the relationship between other uses and the sociolinguistic groups. The results show that the first component explains 41.6% of the information and that the first two components account for 78.6% of the information sought (Fig. 2). The correlation circle revealed that fodder use was positively correlated with the first axis, and commercial use was negatively correlated with the same axis (Fig. 2a). The mystical-religious and medicinal uses were positively correlated with the second axis. The projection of the sociolinguistic groups in the first two axes shows that the Nago use far more common bean in the fodder while the Fon and Adja sociolinguistic groups use it more in medicine (Fig. 2b). Similar trends have been observed with the use diversity values (UD) of common bean which showed higher proportion of fodder (UD = 0.25) for Nago sociolinguistic group and medical (UD = 0.11) for Adja sociolinguistic group (Table 6). On the other hand, Mahi sociolinguistic group use common bean more for mystical-religious purposes, and the Idaatcha and Ifé sociolinguistic groups and a lesser extent the Tchabé sociolinguistic group use it in commercial purpose (sale of seeds or seeds transformed in donuts) (Fig. 2b). However, higher use diversity values (UD = 0.04) of common bean for mystical-religious purposes have been showed for the Ifé sociolinguistic group (Table 6).

### Medicinal uses

Several common bean parts are involved in folk medicine in the central region of the Republic of Benin (Table 7). Results showed that common bean leaves were the most used part by surveyed farmers for diseases treatment (FL = 83.3%), followed by roots (FL = 20.8%), and seeds (FL = 8.3%). The medicinal uses of common bean have been very diverse. The leaves of *Kpankoui rouge*, common bean folk variety, were frequently used by the Adja sociolinguistic group to treat wounds (FL = 19.2%) and babies who cannot be breastfed (FL = 25%). While roots and seeds of this common bean folk variety were used by the Adja sociolinguistic group for difficult childbirth (FL = 25%) and pharyngitis (FL = 25%) respectively. The leaves of *Séssé* landrace were involved in the treatment of sterility (FL = 4.5%) by the Idaasha sociolinguistic group and fever (FL = 44.1%), bee stings (FL = 5.6%), and bad body odor (FL = 5.6%) by the Fon sociolinguistic group. The seeds and roots of *Séssé* landrace were used against late umbilical cord removal in newborns (FL = 5.6%) and obesity (FL = 11.1%) by the Fon sociolinguistic group. Vaginal infection (FL = 11.1%) and bee stings (FL = 5.6%) were also treated with leaves of *Akpakoun vovo* folk variety by the Fon sociolinguistic group.

**Table 7:** Common bean folk varieties used to treat diseases in function of sociolinguistic groups

Organ	Folk varieties	Purpose of use	Processing method	Form of use	Ethnic groups	Fidelity level (%)
Seed	Kpankoui rouge	Pharyngitis	Burn the seed and mix with palm oil and salt	Lick the powder	Adja	25
	Séssé	Rapid umbilical cord removal in newborns	Crush seeds and mix with seasoning cube (Maggi) and water	Pass the mixture on the umbilical cord	Fon	5.6
Leaves	Kpankoui rouge	Babies who cannot breastfeed	Boil the leaves with water	Make the baby drink the liquid	Adja	25
	Kpankoui rouge	Wounds	Crush the leaves and collect the juice	Put the juice in the wound	Adja, Idaasha, Mahi	19.2
	Akpakoun vovo	Vaginal infection	Crush the leaves and mix with water	Take a bath with the liquid	Fon, Mahi	11.1
	Séssé	Bad body odor	Infuse leaves in water	Take a bath with the liquid	Fon	5.6
	Akpakoun vovo, Séssé	Bee stings	Crush the leaves	Apply on the bee sting	Fon	5.6
	Sessé	Sterility	Burn the leaves	Drink with porridge every morning	Idaasha	4.5
	Séssé	Fever	Crush the leaves and roots in water	Take a bath with the liquid	Fon, Ifé, Nago, Tchabé	44.4
Roots	Séssé	Obesity	Crush the leaves and roots	Drink the liquid	Fon	11.1
	Kpankoui rouge	Difficult childbirth	Crush the roots and mix with water	Drink the liquid	Adja	25

### Mystical-religious uses

**Fig. 3:**
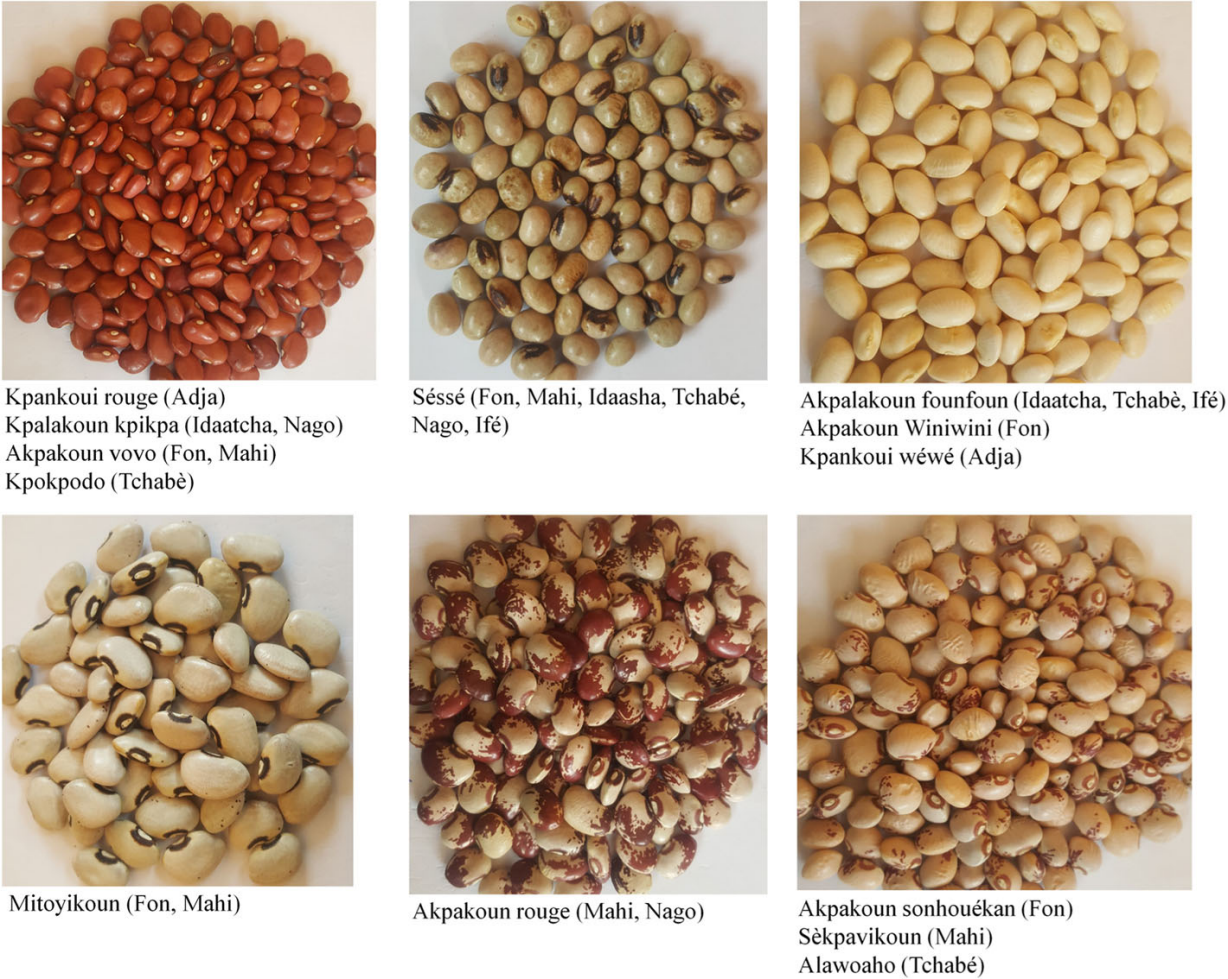
Seeds of common bean landraces used by farmer in central Benin for their medico-magical properties

At least one mystical-religious use of common bean folk varieties was noted through the sociolinguistic groups of the study area, except the Tchabé sociolinguistic group (Table 8). Nine common bean folk varieties (*Mitoyikoun, Akpakoun rouge, Kpalakoun kpikpa, Kpankoui rouge, Akpakoun vovo, Akpakoun wéwé, Akpalakoun founfou, Séssé, Sonouhoué*) were considered by farmers as magical plants which have supernatural properties (Fig. 3). *Akpakoun wéwé*, *Mitoyikoun*, *Akpakoun rouge*, *Sessé*, *Kpankoui rouge,* and *Akpakoun vovo* folk varieties were mentioned to be used by the Mahi, Fon, and Adja sociolinguistic groups for protection of fields (29.1% of responses), homes (20.7% of responses), pregnancies (4.2% of responses), and persons (8.3% of responses) against evil spirit attacks. Seeds of *Akpakoun wéwé*, *Akpalakoun founfou*, and *Kpalakoun kpikpa* are respectively used by farmers of the Fon, Ifé, and Idaasha sociolinguistic groups for traditional ceremonies (Table 8). Roots and seeds of *Séssé* landrace were respectively used for bewitchment treatment (4.2% of responses) in the Fon sociolinguistic group and ceremonies of twins (8.3% of responses) in the Nago and Ifé sociolinguistic groups. The seed of *Séssé* landrace were also used by the Ifé sociolinguistic group in their traditional dance, namely Guèlèdè (4.2% of responses), and by the Idaasha sociolinguistic group to spiritually fight their enemies (4.2% of responses). In the Mahi sociolinguistic group, leaves of *Sonouhoué* folk variety were used for love potions (4.2% of responses).

**Table 8:** Common bean folk varieties used as mystical-religious plants

Roles	Purpose of uses	Folk varieties	Organ	Use	Ethnic groups	Percentage of responses
Protection against evil spirits	Fields protection	Akpakoun wéwé, Mitoyikoun	Whole plant	Planted in the fields	Mahi, Fon	29.1
	Homes protection	Akpakoun rouge, Sessé	Whole plant	Sow next to the houses	Mahi	20.7
	Pregnancy protection	Kpankoui rouge	Leaves	Triturate the leaves in water, then wash with each 3 months so three times before delivery	Adja	4.2
	Protection against enemies attacks	Akpakoun vovo	Whole plant	Go naked towards the plant in the middle of the night and praise the plant and ask for protection against your enemies. Then take the leaves with which you shower after infusion	Mahi	8.3
Treatment of supernaturally caused illnesses	Bewitchment	Séssé	Roots	Triturate the roots in a little water and add the palm kernel oil and drink the potion	Fon	4.2
Traditional ceremonies	Offering to certain deities	Akpakoun wéwé	Seeds	Seeds are prepared and offered to the deities during the rites	Fon	4.2
	Hunters’ ceremonies	Akpalakoun founfou	Seeds	Use to attract animals to hunters	Ifé	4.2
	Twins ceremony	Sessé	Seeds	Seeds are prepared and served to the twins and the family concerned with palm oil at the end of the ceremony	Nago, Ifé	8.3
	Traditional family ceremony	Kpalakoun kpikpa	Seeds	Seeds are cooked with Kersting’s groundnut and served to the guests	Idaasha	4.2
	Guèlèdè traditional dance (ancestor cults)	Séssé	Seeds	Seeds are prepared and served to the followers which helps them to have a good memory	Ifé	4.2
Spiritual warfare	Fight his enemies	Séssé	Seeds	At the ceremony the prepared beans are delivered to the fetish with the name of the enemy	Idaasha	4.2
Love potion	Bring a person to love you	Sonouhoué	Leaves	The leaves are mixed with spider eggs and the last drops of human urine wanting to be loved. The juice obtained is applied to the eyes of the bewitched person.	Mahi	4.2

### Common bean religious prohibitions

In the study area, few farmers (16.8%) reported common bean religious prohibitions. Except the Adja sociolinguistic group, several taboos regarding *Séssé* landrace were inventoried across the other groups. The consumption of *Séssé* landrace was indicated as forbidden for followers of Ogun (deity of fire and war) and Xevioso (deity of lightning), deities the in Idaasha, Nago, and Ifé sociolinguistic groups. *Séssé* landrace was also forbidden for followers of Shango deity in the Tchabé, Nago, Mahi, and Fon sociolinguistic groups. In the Nago and Ifé sociolinguistic groups, followers of Edjo Alowakoyo and Edjo Oko deities (snake deities) do not eat *Séssé* landrace. In the Nago sociolinguistic group, it is forbidden for menstruating women to enter the field of *Sessé* to avoid low yield. Moreover, the study revealed that in the Nago sociolinguistic group, it is forbidden to prepare *Séssé* landrace during the dry season to avoid attracting misfortune on one’s children. In the Idaasha sociolinguistic group, it is forbidden to cultivate *Séssé* landrace close to a voodoo temple because the plant can drive the spirits of the temple out.

### Effects of socio-demographic characteristics on the use of common bean

Multinomial logical regression analysis revealed that the level of education of surveyed farmers positively and significantly influences the medicinal use of common beans (Table 9). However, the commercial use considerably depended on the age of farmers. Moreover, women were strongly more involved in commercial use of common bean than men. The age of surveyed farmers also affected mystical-religious use of common beans. Experience of farmers in common bean production did not relate with its diversity of use (Table 9). Moreover, no significant differences were observed between socio-demographic characteristics of respondents and fodder use of common bean. The pseudo *R*^2^ value of 0.08 indicates that 8% of the variations in common bean use are explained by the independent variables included in the regression model, while the Chi-square value of 37.94 is likely highly significant (*p* <  0.001), suggesting a strong explanatory power of the model.

**Table 9:** Determinants of diversity of use of common bean landraces in central Benin

Explanatory variables	Category of use							
	Medicinal		Commercial		Mystical-religious		Fodder	
	Coefficient	*p* value	Coefficient	*p* value	Coefficient	*P*-value	Coefficient	*p* value
Age	0.011	0.687	0.045**	0.036	0.531*	0.061	0.322	0.426
Sex	0.481	0.492	− 0.895**	0.037	0.213	0.739	0.765	0.491
Education	0.765**	0.037	− 0.408	0.353	− 14.354	0.982	− 14.472	0.989
Experience	− 0.035	0.550	− 0.045	0.324	0.013	0.819	− 0.012	0.888
Constance	− 2.000	0.136	− 1.514	0.145	− 3.993	0.005	− 3.970	0.058
Basic category	Food							
Number of observation	164							
LR Chi 2 (40)	37.94							
− 2 log-likelihood	− 208.51							
Prob > Chi 2	0.001							
Pseudo *R*^2^	0.08							

**, * significant at 5% and 10% probability level respectively

## Discussion

Our study showed that in the study area, each common bean landrace has a local name by which it is identified as a unit of diversity by farmers. Through the surveyed sociolinguistic groups, 26 folk varieties have been found, which could indicate the genetic diversity of common bean in the study area. Considered as an integral part of farmers decision of maintenance, management, and exchange of landraces [32], our results showed that folk taxonomy and nomenclature of common bean landraces are based on morphological, agronomic, and use values characteristics of seeds. These characteristics used for identification of the different common bean landraces are heritable, reflecting the consistence of this folk taxonomy. Similar results have been found by Rengalakshmi [33] which reported that the Malayali tribal farmers of Kolli Hills living in India classify landraces of millet on the base of the morphological, gastronomic, and functional characteristics. The folk taxonomy of common bean landraces in the study area has a hierarchical structure with a low level of classification. Indeed, according to ethno-taxonomic system described by Berlin [34], only two hierarchy levels of common bean classification (varietal and sub-varietal) have been found in all sociolinguistic groups. For example, in the Fon sociolinguistic group, the generic name *Akpakoun* is subdivided into six infra-specific common bean taxa (*Akpakoun wéwé, Akpakoun vovo, Akpakoun wiwi, Akpakoun sonhouékan, Akpakoun wéwé winiwini, and Akpakoun wéwé gaga*). These folk common bean taxa recorded reflect the cultural value and the diversity of common bean in this sociolinguistic group. The diversity of local names given to common bean folk varieties is the evidence for the long establishment of beans as food crop in the region. Similarly, to southern Ethiopia farmers [35], most farmers in the central region of the Republic of Benin often used seed color and seed size in naming common bean landraces. Although the names differed from one language to another, similar results have been reported in the Republic of Benin on cowpea [36], fonio [37], and sorghum [38]. The analysis of the meaning of local names given to common bean landraces in the study area confirms the existence of various scenarios (unexplained names, synonymy, and even local names used by different sociolinguistic groups). This is common to the vernacular nomenclature of many legumes in the Republic of Benin such as the cowpea [36] and Kersting’s groundnut [39]. The most important morphological trait used in common bean folk taxonomy in the study area was the seed coat color, which is still the most used as marker in studies on assessment of common bean diversity [40]. Hence, selection based on common bean seed coat color will have a definite role in the framework of on-farm conservation of this legume in central Benin. In fact, it is known that knowledge in folk taxonomy makes genetic resources collection and conservation simple, practical, and very objective [21].

The production of common bean landraces by all sociolinguistic groups in the central region of the Republic of Benin reflects the cultural importance and the maintenance of this legume in traditional agriculture. On the base of seed’s morphological description characteristics and subject to synonymy, we recorded 12 common bean landraces in the study area. This diversity is higher than those found in southern Ethiopia (6 landraces) by Asfaw et al. [35], and lower than those found by Martin and Adams [41] in northern Malawi (15 landraces). The results showed that classification of common bean landraces varied from one sociolinguistic group to another. Therefore, several folk varieties could be attributed to a single landrace, and many landraces could have a same name. This situation could contribute to under or over-estimate of the diversity of this legume in the study area. So, to avoid redundancies and optimizing the efficient conservation and sustainable use of common beans, agro-morphological and molecular characterization is recommended.

This study showed that common beans are multipurpose species in the central region of the Republic of Benin, and all part (leaves, seeds, roots) are exploited. Through the sociolinguistic groups in central Benin, farmers produce common beans mainly for food. This is not surprising because in most cases farmers mostly grow the species for their dietary needs [42]. Other fodder, medicinal, and mystical-religious uses were notified by farmers. They indicate their good awareness of common bean’s potential. However, common bean can have a veterinary use as is the case in southern Italy, where the decoction of common bean seeds is claimed to be a galactophorous for cows [43]. The fact that the Idaasha, Ifé, and Tchabé sociolinguistic groups were more oriented towards the marketing of the common bean is not surprising. Indeed, these sociolinguistic groups are the descendants of the Yorouba people from Nigeria which are traditionally known as traders in their host countries [44]. Fodder use of common bean is done by many farmers in some regions [45]. In Benin, the Nago sociolinguistic group is a sedentary people who practice the breeding of small ruminants [46], which can justify the fact that they were more involved in fodder use of common bean. Knowing that, endogenous knowledge is cultural and, thus, variable from one sociolinguistic group to another [47], the medicinal use of common beans by the Adja and Fon sociolinguistic groups could be explained by the fact that medicinal properties of this legume is based on indigenous customs and practices. In fact, Koutchade et al. [48] shown that the Adja and Fon sociolinguistic groups know more plants and recipes for treating childhood diseases than the other sociolinguistic groups.

Common bean is an important medicinal plant throughout the world. It is used by farmers for jaundice treatment in southern Ethiopia [49], as in India where rice landrace, namely Bora, was used to treat this disease [50]. Ajao et al. [19] have found that common bean enter in rheumatism treatment in Nigeria. In fact, common bean have some bioactive components related to health benefits [51, 52] that have shown to treat diabetes [14]. The chemoprevention properties of common bean against cancer were demonstrated by Metha et al. [53]. Scientific evidence also shows that this legume may act as an effective anti-inflammatory [13], analgesic [54], antifungal [55] and antibacterial [56] functional food. Therefore, exploitation of the potentials of common bean landraces in traditional medicine must be promoted. The treatment of obesity reported by farmers, confirmed by some scientific studies [15, 16], gives a scientific support to indigenous knowledge in the identification of plants for treating diseases [26]. However, gaps in knowledge still remain on the medicinal properties of common beans responsible for the curing of ten other diseases listed in the study area. It is therefore important to verify the statements of farmers in the central region of the Republic of Benin on the medicinal properties of the *Kpankoui rouge*, *Séssé*, and *Akpakoun vovo* folk varieties according to different treated diseases.

Similarly, to surveyed farmers in the study area, many native peoples used plant in ceremonial and spiritual ritual events from immemorial time [57]. For example, common bean seeds were used as sacramental objects in ceremonies and rituals for prediction with prayer in Romania [17]. As revealed by farmers in the study area, Crosson [58] notified that dried beans can be used for protection, love potion, and to fight evil spirits. Similarly, to the Idaasha sociolinguistic group, the Yoruba people also use common bean seeds to serve for invited guests [59]. The use of this legume as offering during ritual to deities by the Fon sociolinguistic group was also observed in the Maya people of the peninsula of Yucatan which used common bean during rain ritual [60]. Moreover, beans are used during festivals of new yams as sacrifice to the fetish of yams [61]. All these mystical uses of common bean landraces show the potentiality of cultural approach for the conservation of this legume in the study area. In fact, it is known that integrating technical expertise and cultural practices of local communities permit an efficient on farm conservation [62].

Some taboos are associated to the consumption of certain common bean landraces for followers of deities in the study area, and breaking the ban is often followed by supernatural penalty [63]. This result is in line with the view of Missinhoun et al. [6] which notified that in the Hlagba-Zakpo village of southern Benin, beans are prohibited for followers of Lègba deity. Moreover, in Ghana, certain people are instructed by the gods not to eat beans because it causes stomach disorders [64]. Similarly, food taboos related to bean consumption were observed in Mid-West Nigeria [65]. Our results showed that *Séssé* landrace was a common taboo for followers of all the deities listed by farmers; this could be explained by the fact that all these deities were derived from the Yoruba region. These religious prohibitions of *Séssé* landrace could cause its disappearance in the study area. It is therefore important to evaluate its distribution and extent in the Republic of Benin in order to develop a strategy for the conservation of this common bean landrace.

Among the determinant of common bean use, the results have shown that gender influences its commercial use. Indeed, several studies have shown that women are more involved in the common bean trade [45, 66, 67]. The significant increase of commercial use of common beans with increasing informant age was contrary to the results of Mbitsemunda and Karangwa [68] which found that age negatively and significantly influenced common bean commercial use. Similarly, Birachi et al. [69] indicate that younger farmers are more likely marketing beans than older farmers. This result could be explain by the fact that common bean production is principally done by farmers of a certain age, and Ngoh et al. [70] shown that when farmers produce more common beans, they are more likely to participate in its commercialization. As for the medicinal use of common beans, it is influenced by the level of education of farmers. This result confirms the importance of an academic education level on the use of medicinal plants shown by Oldendick et al. [71], Duru et al. [72], and Ghaedi et al. [73]. The age of surveyed farmers also significantly affected mystical-religious use of common beans. This is not surprising because it is known that the knowledge of useful plant species is higher in elderly than with younger people [74]. This is probably due to the accumulation of ethnobotanical knowledge through their life [75, 76].

Our findings showed that common beans are well integrated in local traditions, and some socio-demographic characteristics of farmers influence their uses in the central region of the Republic of Benin. So, the conservation of common bean diversity in the study area requires the maintenance and preservation of traditional knowledge associated to this legume through educational and cultural programs connected to conservation of varietal diversity. Moreover, breeding of erected common bean to overcome availability of staking materials, which is the main production constraint that producers face, was preconized by Missihoun et al. [6] for the promotion of common bean in the Benin Republic.

## Conclusion

Folk nomenclature and taxonomy of common bean folk varieties documented in the study area were mainly based on seed traits and could help for communication among researchers, extension agents, and farmers as part of in situ conservation programs of this legume in the central region of the Republic of Benin. This study showed a diversity of its use, depending on the socio-linguistic groups existing in the central region of the country. Common bean seeds are consumed by all of the sociolinguistic groups, and the different parts of the plant are used by farmers in folk taxonomy to treat several diseases. Some common bean landraces were considered by farmers as having mystical-religious properties. Traditional values associated with landraces of this legume increase their chance of survival in the farming system. However, *Séssé* landrace is prohibited for some followers of deities which can lead to its disappearance. To promote the conservation and sustainable use of common bean landraces in the central region of Republic of Benin, in situ and ex situ conservation strategies should be considered.

## Abbreviations

*p*: *p* value
